# Effect of Biochar Addition on Mechanical Properties, Thermal Stability, and Water Resistance of Hemp-Polylactic Acid (PLA) Composites

**DOI:** 10.3390/ma15062271

**Published:** 2022-03-19

**Authors:** Mariem Zouari, David B. Devallance, Laetitia Marrot

**Affiliations:** 1 Innorenew CoE, Livade 6, 6310 Izola, Slovenia; devallance@innorenew.eu (D.B.D.); laetitia.marrot@innorenew.eu (L.M.); 2 Faculty of Mathematics Natural Sciences and Information Technologies, University of Primorska, Muzejski Trg 2, 6000 Koper, Slovenia; 3 Andrej Marušič Institute, University of Primorska, Muzejski Trg 2, 6000 Koper, Slovenia

**Keywords:** biochar, polylactic acid, hemp fibers, mechanical properties, thermal stability, water resistance

## Abstract

The present study investigated the effect of biochar (BC) addition on mechanical, thermal, and water resistance properties of PLA and hemp-PLA-based composites. BC was combined with variable concentration to PLA (5 wt%, 10 wt%, and 20 wt%) and hemp (30 wt%)-PLA (5 wt% and 10 wt%); then, composites were blended and injection molded. Samples were characterized by color measurements, tensile tests, thermogravimetric analysis (TGA), differential scanning calorimetry (DSC), and water contact angle analysis. Experimental results showed that adding 5 wt% of BC enhanced the composite’s tensile modulus of elasticity and strength. Hence, the use of optimized loading of BC improved the mechanical strength of the composites. However, after BC addition, thermal stability slightly decreased compared with that of neat PLA due to the catalytic effect of BC particles. Moreover, the water-repelling ability decreased as BC content increased due to the specific hydrophilic characteristics of the BC used and its great porosity.

## 1. Introduction

The increase of environmental awareness, concerns related to ecological impact, waste storage and accumulation, and the depletion of synthetic resources have raised a growing interest in eco-friendly materials derived from renewable resources. Indeed, to serve specific applications and respect the current environmental challenges, novel sustainable composites need to be invented.

Specifically, polymeric composites reinforced by plant fibers demonstrate interesting properties such as high strength, low weight, and reduced environmental impact [1]. Polylactic acid (PLA) is a biobased aliphatic polyester produced by fermentation of natural glucose-rich resources such as corn, cassava, and sugarcane. The PLA matrix has demonstrated distinctive compostability, rigidity, and processability [2,3]. The global demand for PLA continues to grow and is forecasted to increase 18.1% annually until 2028 [4]. PLA is used in various applications such as food packaging, 3D printing filaments, textiles, and biomedical tools [4]. PLA use is limited by its price, degree of crystallization, inherent brittleness [5], and its relatively low glass transition temperature that negatively affects thermal resistance. Reinforcing PLA with plant fibers can enhance the resulting mechanical [6] and thermal properties [7]. In particular, long hemp fibers were reported as a potentially effective reinforcement in PLA-based composites that showed improved features [8,9,10]. However, the current high price of PLA and other shortcomings, such as thermal, mechanical, and moisture resistance remain key challenges that have limited the use of plant fibers reinforced-PLA composites.

Additives (i.e., fillers) can be used to reduce the price of PLA. Biochar (BC) is an interesting candidate to reduce prices and improve the performance of PLA composites. BC is a carbon-rich material derived from biomass pyrolysis at high temperatures in an oxygen-free atmosphere [11]. BC is a renewable and abundant material prepared from many types of biomass, including waste and low-value feedstocks [12]. BC recently emerged as filler in polymeric composites, including thermoplastics and thermosets [13,14]. The attractiveness of BC lies in its superior characteristics (depending on pyrolysis conditions), such as hydrophobicity [15], electrical conductivity [16,17], high surface area, and porosity [18].

Aup-Ngoen et Noipitak [19] evaluated the influence of BC reinforcement (0.25 wt%) prepared from different agricultural biomasses (cassava rhizome, durian peel, pineapple peel, and corncob) on PLA-BC composites. Their results indicated that BC powder derived from cassava rhizome increased the composite’s elastic modulus and impacted energy by 20.7% and 76%, respectively, compared to neat PLA. Similarly, Nan et al. [20] investigated the mechanical properties of polyvinyl alcohol (PVA) thin films reinforced with hardwood BC. They found that the tensile modulus increased by 129%, 271%, and 429% when adding 2 wt%, 6 wt%, and 10 wt% of BC, respectively, compared to pure PVA. DeVallance et al. [21] studied the effect of BC incorporation in wood-polypropylene (PP) composites. They concluded that samples reinforced with 5 wt% and 40 wt% BC exhibited higher tensile strength and water resistance compared to wood-PP composites. Das et al. [22] used waste wood BC in PP-30 wt% wood flour bio-composites. They reported the positive effect of BC addition on mechanical and thermal properties, with 24 wt% addition of BC exhibiting the best properties.

To date, most research focused on adding BC to the polymer material, not fiber-reinforced polymer composites. Few studies addressed the effect of plant fibers and BC reinforcement in polymer-based materials [23]. However, in this study, we investigated the effect of beech-derived BC addition on the properties of PLA composites reinforced with short hemp fibers. Composites were prepared by blending of PLA-hemp fibers (0 and 30 wt%)-BC (0, 5, 10, and 20 wt%) mixtures followed by injection molding of tension test samples. The influence of adding BC on the color, mechanical properties, thermal stability, and water repellency of the resulting composites was studied and thoroughly discussed.

## 2. Materials and Methods

### 2.1. Materials and Reagents

Biochar (BC) produced at 550 °C using beechwood (labeled as Q004) was provided by Profagus GmbH (Bodenfelde, Germany). Polylactic acid (PLA) pellets (Inzea F38) dedicated for injection molding applications were purchased from Nurel biopolymers. The PLA used in this study had a bio-based content of 70%, a melt flow index of 1.8 g/10 min (190 °C/2.16 Kg), and a density of 1.23 g/cm^3^. Hemp fibers (Cannabis Sativa, dioecious Hungarian variety Tisza) were supplied by Hempson, and their properties were characterized in our previous study [10]. Sodium Dodecyl Sulfate (purity = 99.0%) purchased from Sigma Aldrich was used for particles size analysis. Technical nitrogen for thermogravimetric analyzer and nitrogen and carbon dioxide gases with a purity of 5.0 were used for physisorption analysis.

### 2.2. Preparation and Characterization of the Fillers

The characterization of the BC powder included proximate analysis using a thermogravimetric analyzer (LECO TGA801) following ASTM-D7582 procedures (LECO corporation, Saint-Joseph, MI, USA) to determine the moisture, volatile, ash, and fixed carbon contents. Particle size was measured by laser diffraction using a Horiba Scientific LA-960A2 analyzer (HORIBA, Kyoto, Japan) with the refractive index set to 1.92. For homogeneous particle dispersion during the particle size testing, sample powder was first mixed with 2 mL of distilled water and 2 mL of 20% Sodium Dodecyl Sulfate solution and ultrasonicated for 1 min before the measurement. Additionally, the surface area and pores volume were determined by physisorption analysis (Anton Paar Quantachrome Instruments, Boynton Beach, FL, USA) using nitrogen and carbon dioxide gases.

Hemp fibers were manually cut into short pieces with a custom-made guillotine, and their length was evaluated using a digital microscope Keyence VHX-6000 (Keyence corporation, Itasca, IL, USA).

### 2.3. Preparation and Characterization of the Composites

Seven composite formulations were prepared using PLA, BC, and hemp fibers. The formulation’s weight ratios are listed in Table 1. The manufacturing process was performed using two steps. The mixtures were blended using a small mixer (Thermo Scientific™ HAAKE™ Rheomex PTW 16 OS, Waltham, MA, USA) at 190 °C for 3 min using a screw rotation speed of 400 rpm. The blends were then collected, cooled at ambient temperature, and ground into 4 mm particles using a cutting mill grinder (Fritsch PULVERISETTE 25/19, Amberg, Germany). Dog bone-shaped, tensile test specimens were prepared according to ISO 527 standard for plastics and plastic composites. Composites were fabricated using the ground particles through injection molding (Thermo Scientific™ Minijet Pro, Waltham, MA, USA) at 190 °C under a 900 bar pressure. For each formulation, five dog bone-shaped, tensile test specimens were prepared according to ISO 527 standard for plastics and plastic composites.

The color of the composites was investigated, and measurements were performed using a Spektromaster 565-45 spectrophotometer (Erichsen, Hemer, Germany) according to the CIELAB color system. The color measuring instrument has the following characteristics: light source D65; standard observer 10°; spectral range 400–700 nm; spectral resolution 10 nm; aperture size 11 mm; and fixed geometry of measurement 45°/0°. Three repetitions were performed per composite formulation, and the average values were reported.

The mechanical properties of the produced composites were evaluated using tensile strength testing according to ISO 527 over a 50 mm gauge length. Loading was applied at a rate of 5 mm/minute crosshead movement using a Zwick Roell 50KN universal test machine (UTM) (ZwickRoell, Ulm, BW, Germany). Displacement was measured using the UTM crosshead travel. Five repetitions from each formulation were tested. The tensile strength, tensile modulus of elasticity, and strain at failure were determined using test Xpert III software.

Thermogravimetric (TGA) analysis was performed using TGA 5500 (TA Waters Instruments, New Castle, DE, USA) using 15 mg samples (three repetitions per composite formulation) placed on platinum pans. The samples were heated at a 10 °C/min rate, and the weight loss was recorded over a temperature range from 25 to 500 °C under 25 mL/min flow of nitrogen gas. Trios software was used to determine the weight derivatives and extract data for T_5%_ and T_10%,_ which correspond to temperatures at which 5% and 10% weight loss was achieved, respectively. Additionally, the temperature at the maximum rate of sample degradation (T_max_) was recorded as the highest point in the peak appearing in the derivative curve (dTG). The final residue percentage at 500 °C was also determined.

Differential Scanning Calorimetry (DSC) analysis was carried out using DSC 25 (TA Waters Instruments, New Castle, DE, USA) using 6 mg samples (three repetitions per composite formulation) placed in aluminum pans sealed with aluminum lids. The samples were subjected to the following heat-cool-heat cycles in a nitrogen gas flow of 50 mL/min:

- First heating from 30 to 220 °C at 10 °C/min.
- Cooling from 220 to −30 °C at 10 °C/min.
- Second heating from −30 to 220 °C at 10 °C/min.

The thermal parameters, i.e., glass transition temperature (T_g_), cold crystallization temperature (T_cc_), melting temperature (T_m_), crystallization temperature (T_c_), and enthalpies, were determined.

The degrees of crystallinity of pure and reinforced PLA were calculated using Equation (1):

(1) $$X\%=\frac{\Delta Hm-\Delta Hcc}{\Delta H_{m}^{0}}\times 100$$

where Δ*Hcc* and Δ*Hm* are representatives of the cold crystallization and the melting enthalpies, respectively, and $\Delta H_{m}^{0}$ is the melting enthalpy of 100% crystalline PLA, which is equal to 93.7 J g^−1^.

The surface hydrophobicity of the composites was evaluated by measuring the dynamic contact angle using an optical tensiometer Attension Theta Flex Auto 4 system (Biolin Scientific, Gothenburg, Sweden) equipped with a 3D topography module. Measurements were made at ambient temperature using a sessile drop test method. For each measurement, a 4 µL distilled water drop was placed on the specimen. Ten contact angle measurements were performed on each specimen, and three specimens were tested per formulation.

The wetting energy (*Ewet*) was determined using the following Equation (2) [24].

(2) Ewet=γ×cosθ

where *θ* is the WCA (at 30 s) and *γ* is the surface tension of the liquid used during the experiment, which is distilled water in our study (*Ewet* of distilled water = 72.8 mJ/m^2^).

Immersion tests were conducted according to ASTM D 570-98 to assess the composites’ water absorption (WA) behavior. The different samples were oven-dried at 50 °C for 24 h and cooled to room temperature in a desiccator to avoid atmospheric humidity absorption. Then, the samples were weighed using an analytical laboratory scale (kern ABT 220-5DNM). Subsequently, the samples were immersed for 24 h and 48 h in distilled water at ambient temperature. After the defined immersion time, the samples were taken out of the water, wiped, and weighed. The *WA* percentage was calculated using Equation (3):

(3) $$WA\%=\frac{mt-m0}{m0}\times 100$$

where m0 and mt represent the specimen weight at *t* = 0 (initial dry weight) and after immersion for *t* time (24 h and 48 h), respectively. Presented results are the average of three repetitions per composite formulation.

## 3. Results and Discussion

### 3.1. Characteristics of the Fillers

The characteristics of the BC used during this study are summarized in Table 2.

The proximate analysis results reflect a typical composition of wood-derived BC, and values for volatiles and fixed carbon (FC) are comparable with those reported by previous research [25] for pinewood (22% and 73.9% for volatiles and FC, respectively).

D_10_, D_50_, and D_90_ represent the particles’ diameter at 10%, 50%, and 90% of the cumulative size distribution, respectively. The mean particle size was 75 ± 4 µm. Overall, the BC powder was fine, as reflected by the micro-sized particles. The small size of the filler particles is favorable for a uniform distribution within the composites when introduced into the polymeric matrix.

BC’s surface area and pore volume are critical properties that influence performance in different applications. The porosity of carbonaceous materials is commonly composed of micro-pores (0–2 nm in width), meso-pores (2–50 nm in width), and macropores (width > 50 nm) [26]. The physisorption analysis was conducted using nitrogen and CO_2_ gases to provide a complementary description of the sample’s pores distribution. In fact, nitrogen gas usually determines the outer surface and pores larger than 1 nm because the nitrogen’s access to narrower pores is limited; thus, it does not give a complete estimation of the sample’s porosity. Whereas CO_2_ specifically gives information about the microporosity of the material [27]. The surface area and porosity of the used BC are higher compared to those of BC derived from applewood: 37.24 m^2^/g and 0.012 cc/g for surface area (SA) and total pores volume (TPV) from nitrogen gas measurements, respectively and 9.33 m^2^/g and 0.0016 cc/g for microporous SA and microporous TPV from CO_2_ measurements, respectively [28]. For pinewood BC prepared at similar temperatures, Meléndez et al. [29] reported slightly higher SA from nitrogen physisorption analysis equal to 267 m^2^/g; however, they obtained lower values for microporous SA and TPV from carbon dioxide physisorption (159 m^2^/g and 0.074 cc/g respectively).

The representative average hemp fibers length was 1.5 ± 0.3 mm. Given the length, these fibers would be considered to be “short fibers” for the purpose of reinforcing composites [30].

### 3.2. Color Measurements of the PLA-Based Composites

**Figure 1:**
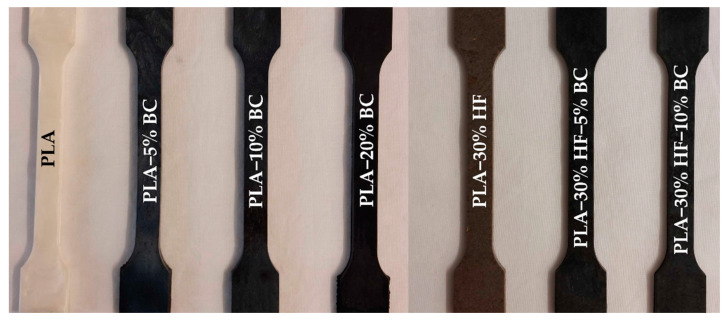
PLA and reinforced PLA composites.

One of the challenging requirements for reinforced polymeric composites is to obtain a uniform appearance. The extent of dispersion of the added fillers affects the final material’s morphology, opacity, and color. Figure 1 presents the effect of biochar (BC) addition on the optical features of the composites. A drastic change in the appearance of PLA was visible from the addition of 5 wt% BC. These visual observations were further supported by the CIE Lab test results listed in Table 3.

CIE a* and CIE b* values recorded for neat PLA show that PLA*’*s color shade tends toward green (negative CIE a*) and yellow (positive CIE b*). The lightness (CIE L*) of PLA reduced sharply after adding BC particles and continued to decrease as the BC content increased because of BC*’*s black color. Overall, we observed that all the BC-containing specimens tended to have higher greenness and blueness. In contrast, the sample filled only with hemp fibers had higher redness and yellowness represented by positive CIE a* and CIE b*, respectively. Visually, the hemp-PLA sample was characterized by a dark brown color. However, the used hemp fibers had a light brown color typical for natural dry plant fibers. This color change was most likely attributed to the physicochemical modifications during the blending and injection molding at relatively high temperatures (190 °C). Similar findings were demonstrated by Yang et al. [31], who investigated the effect of extrusion temperature on the appearance of wood fibers reinforced-PLA.

### 3.3. Influence of Biochar Content on the Mechanical Properties of the PLA-Based Composites

**Figure 2:**
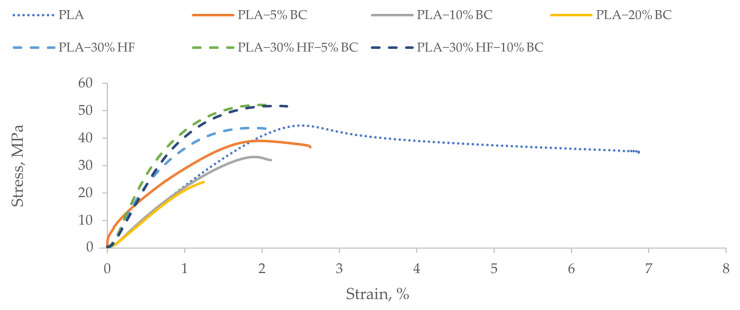
Stress-strain plots of PLA-based composites.

Figure 2 provides the stress-strain curves related to tensile testing of PLA-based composites, and Table 4 gathers the obtained tensile properties.

The PLA manufacturer*’*s technical data sheet listed the tensile modulus of elasticity and strength (determined according to ISO 527-1/-2) as 2300 MPa and 45 MPa, respectively. In this research, the average values of tensile properties found for the neat PLA samples were relatively consistent with the manufacturer*’*s values, with tensile strength being slightly lower. Compared with the neat PLA formulation, the addition of BC at the 5 wt% loading level enhanced the tensile modulus of elasticity (tensile MOE) by 38%. However, the tensile strength (R_m_) decreased slightly (by 5%). However, a further increase in BC content to 10 and 20 wt% led to a successive 21% and 32% reduction in tensile MOE, respectively, and a 16% and 57% reduction in R_m_, respectively when compared to PLA 5% BC. PLA-20% BC samples exhibited the poorest mechanical strength where R_m_ decreased by almost 59% compared to the neat PLA reference. The reduction in properties when the BC content was higher was attributed to the less uniform dispersion of BC within PLA polymers. As the BC content increased above 5%, a higher viscosity was noticed during the processing of the composites in the blending step. For high BC contents, subtle BC aggregates likely appeared, and the consequent low dispersion decreased the interfacial adhesion between the filler and the matrix. Moreover, the aggregations of BC particles would have limited the effective stress transfer between BC particles and PLA chains. Similarly, Moustafa et al. [32] observed aggregation of torrefied coffee powder when loaded at 30% to reinforce poly(butylene adipate-co-terephthalate) (PBAT) matrix. In another study [33], PLA- bamboo-derived BC hybrids were investigated, and results showed that both flexural modulus and strength increased gradually with BC content from 2.5 wt% to 7.5 wt%, then decreased as BC content was raised to 10 wt%. The authors explained that the good compatibility between BC particles and PLA polymers enhanced the extent of interaction between the composite*’*s components which resulted in stabilizing the system and avoiding the de-bonding between filler and matrix during testing. Nonetheless, when BC content was further increased, BC particles tended to agglomerate and form microcracks responsible for a decrease in the flexural and mechanical properties. In a similar experiment, Arrigo et al. [34] found that the tensile MOE of PLA-BC composites increased proportionally with increasing BC loading from 1 wt% to 7.5 wt%. They attributed this increase in tensile MOE to a good filler dispersion within the polymeric matrix, which led to better bonding between the matrix and filler. Similarly, they reported a slight decrease in the R_m,_ which was associated with the porous nature of BC that provides micro-voids inside the composites. Nan et al. [20] also observed a 58%, 75%, and 81% decrement in R_m_ of polyvinyl alcohol (PVA) films when adding 2 wt%, 6 wt%, and 10 wt% of hardwood BC, respectively. They attributed the results to the relatively large BC particles’ size (250 µm) and distribution. Regarding nanosized materials, Paiva et al. [35] also reported a 4.8% decrease in strength when using nanosized carbon tubes (2.5 wt%) for reinforcing PVA composites. In this research, they attributed the reduction in strength to the tendency of the nanoparticles to agglomerate, preventing the uniform dispersion within the matrix.

Therefore, in our study, the reduction in R_m_ in the composites that did not contain short hemp fibers was attributed to the porosity of the used BC (SA and microporous SA equal to 230.8 and 480.0 m^2^/g, respectively) (Table 2), poor dispersion of the BC filler within the matrix, and large distribution of particles size (size ranges from 11 µm to 155 µm as reported in Table 2). For hemp-PLA (PLA-30% HF) specimens, the average tensile MOE and R_m_ increased by 113% and 13%, respectively, compared to neat PLA. Hence, the short hemp fibers provided efficient reinforcement of the polymer matrix. In fact, short fibers demonstrated a good strengthening potential when used as filler in thermoplastic composites [36,37]. In a prior study, Sawpan et al. [38] reported higher values for tensile MOE and R_m_ (approximately 7500 MPa and 66 MPa, respectively) when using 30% hemp fiber loading. Regarding potential reinforcement of the PLA-hemp composites, when 5 wt% BC was added, tensile MOE and R_m_ increased by 13.2% and 11.6%, respectively. One possible justification for the BC*’*s positive effect on both tensile MOE and R_m_ is that the BC may have helped reduce the agglomeration of the hemp fibers, which would normally lead to enhanced overall strength and elasticity of the composite. However, the potential for BC to improve the dispersion of short hemp fibers required further investigation. As the BC content increased to 10 wt%, a slightly lower tensile MOE and higher R_m_ was observed for the hemp-PLA composites. Kumar et al. [23] also reported enhancements in tensile and flexure strength and flexure modulus when adding up to 1.5% of coconut shell BC prepared at 300 °C and 400 °C to bamboo fibers (40 wt%)-polypropylene (PP) composites. In this same research, however, they reported no improvement in mechanical properties when adding up to 1.5% coconut shell, BC prepared at 600 °C and 900 °C. They attributed the positive effect of BC fillers prepared at lower temperatures to surface functional groups (carboxyl and hydroxyl groups) that improved the filler-matrix compatibility and adhesion. Based on their findings, using BC produced at lower temperatures or modifying the carbonization method to result in higher surface functional groups could potentially improve the hemp-PLA composites produced in our research and warrants additional research.

The strain at break values (Table 4) showed a decreasing trend with the increase of BC content which indicates that plastic deformation was lower when compared to neat PLA. The plastic character of neat PLA was shown by its high strain at break (6.8%). With the introduction of BC in PLA and hemp-PLA composites, the composites exhibited more quasi-brittle behavior under the applied force. Ho et al. [33] found similar results as they observed higher plasticity in the PLA and PLA-2.5 wt% BC samples while the PLA- (5, 7.5, and 10 wt%) BC were more brittle. They suggested that BC particles reduced the plasticity and ductility of PLA matrix and provided higher resistance to deformation.

### 3.4. Thermal Properties

#### 3.4.1. Thermogravimetric Analysis (TGA)

**Figure 3:**
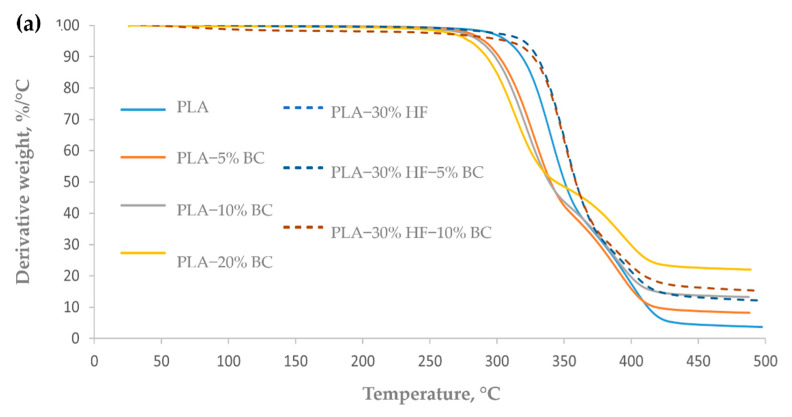

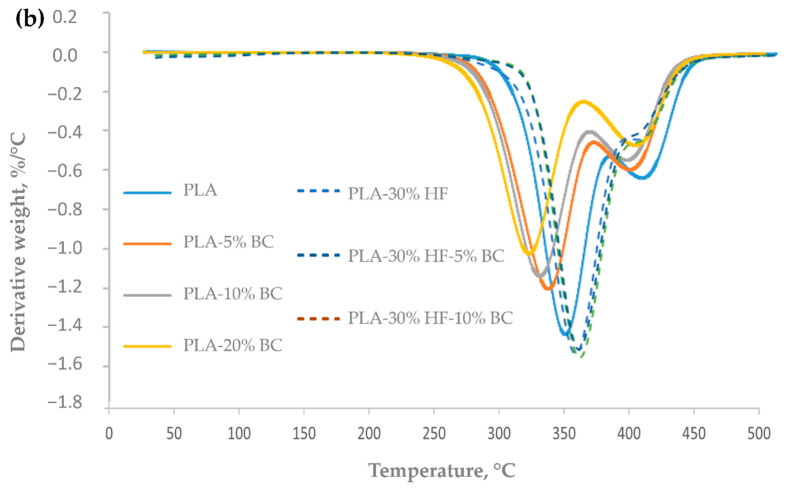
TG (**a**) and dTG (**b**) thermograms of PLA-based composites.

Thermogravimetric analysis was conducted on neat PLA and hemp-PLA composites with and without BC. TGA (TG) and TGA derivatives (dTG) curves of PLA and its composites are represented in Figure 3, and temperatures corresponding to 5% weight loss (T_5%_), 10% weight loss (T_10%_), and to the maximum degradation rate (T_max_) are listed in Table 5, as well as the percentage of final residue at 500 °C.

As shown in Figure 3a, the thermal decomposition began at a lower temperature in the case of the three PLA-BC samples, and accordingly, the peak temperatures of the corresponding dTG curves (Figure 3b), indicating the maximum weight loss rate, was 4.25 °C, 6.69 °C, and 12.23 °C lower for PLA reinforced with 5, 10 and 20 wt% BC, respectively compared to the pure PLA. Hence, after the addition of BC particles, the T_5%,_ and T_10%_ decreased proportionally with the increase of BC percentages, which suggests that BC accelerated the thermal degradation of PLA polymers. Prior research [32] explained that the accelerated polymer degradation when BC was included was mainly related to the catalytic effect of potassium present in BC. Potassium can occur in BC as a component of the ash part. In our study, the proximate analysis revealed an ash content of 1.8% (Table 2) of the used BC. However, the presence of potassium in our BC should be confirmed by other techniques. Arrigo et al. [34] similarly reported that T_5%_ and T_10%_ diminished gradually with the addition of spent ground coffee-derived BC (from 1 to 7.5 wt%) to the PLA matrix. They also verified the presence of K (21.6 wt%) in their BC by mean of Energy dispersive X-ray analysis.

The final residue at 500 °C generated by neat PLA is the lowest value among all the composites tested in this study. The final residual amount (Table 5) increased with the increase of BC content which is due to the accumulation of higher char amount able to persist at high temperatures. Similarly, Angin et al. [39] explained that due to the presence of BC, a compact layer with high char content was formed, and as a consequence, the residual amount increased.

The addition of 30% hemp fibers to the PLA matrix shifted the thermal decomposition of the hemp-PLA composites to higher temperatures compared to the neat PLA. Hemp fibers are mainly composed of cellulose, moreover hemicellulose, lignin, soluble and organic matter [10] that have different thermal resistances. Lignin is reported to slowly decompose from ambient temperature to 900 °C [40], followed by the decomposition of hemicellulose occurring between 220 °C and 315 °C [40], and the decomposition of cellulose between 315 °C and 400 °C [40]. Thus, the improvement in thermal resistance is due to the high cellulose content in hemp fibers. Indeed, Marrot et al. [17] reported a 77–80% *w*/r*w* cellulose of the dry material. Thus, the improvement in thermal resistance is due to the high cellulose content in hemp fibers, which decomposition temperature range is higher than the PLA’s one. The high standard deviations found for PLA-30% HF (for T_5%_ and T_10%_) indicate a non-uniform dispersion of the fibers during the preparation process. Upon incorporation of 5 wt% BC in the hemp-PLA composites, T_5%_ and T_10%_ increased by 4.4 °C and 2 °C, respectively, indicating an enhancement in the thermal resistance. However, a further increase in BC content to 10 wt% resulted in a slight decrease of the degradation temperature points.

#### 3.4.2. Differential Scanning Calorimetry (DSC)

**Figure 4:**
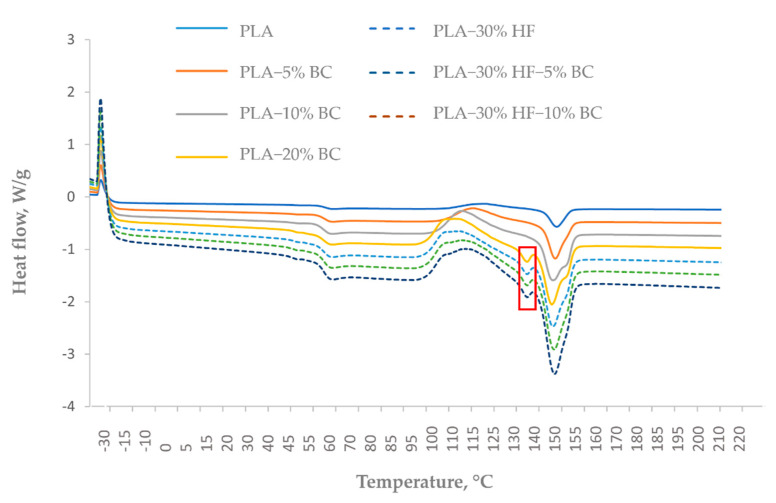
DSC thermogram from the second heating cycle of the PLA-based composites.

The effect of BC additive on thermal transitions of PLA and hemp-PLA composites was evaluated using DSC analysis. The first heating cycle is performed to eliminate the thermal history of the polymeric material [35]. As such, data retrieved from the second heating cycle were considered for discussion. Figure 4 represents the corresponding DSC thermogram corresponding to the second heating cycle. The glass transition temperature (T_g_), cold crystallization temperature (T_cc_), melting temperature (T_m_), crystallization temperature (T_c_), and crystallinity degree (X_c_) of neat and reinforced PLA composites are summarized in Table 6.

Results for PLA-BC samples showed that the addition of 5 wt% and 10 wt% BC did not have a visible effect on the T_g_ of PLA polymer. However, 20 wt% BC decreased the T_g_ value from 58.8 °C to 52.9 °C, which indicates that a high amount of BC increased the mobility of PLA molecular chains. Chen et al. [41] studied the isotherms of PLA reinforced by variable concentrations of recycled grains derived from the distillation of Chinese spirits (from 10 wt% to 50 wt%). Their results suggested that T_g_ diminished with the increase of filler content because of the occurrence of polar groups in the filler*’*s molecules. These polar groups were responsible for high electrostatic repulsions within the composites’ matrixes, which increased the spaces between the polymeric chains of PLA, and in turn, the T_g_ dropped. On the contrary, the addition of hemp fibers did not affect the PLA*’*s T_g_, and the further addition of 5 and 10% BC in the hemp-PLA composites did not lead to a significant decrease of the T_g_.

The broad exothermic peak after the T_g_ corresponds to cold crystallization phenomena. For neat PLA, the cold crystallization peak was very low. However, for the other composites, the corresponding peaks were more visible. In the case of PLA-BC composites, a visible decrease in T_cc_ was correlated with the increasing BC loadings. Arrigo et al. [34] reported similar findings and stated that the BC filler enhanced the degradation of PLA molecules. As a result, the molecular mass of the molecules was lowered, and they became more mobile which favored the appearance of cold crystallization peaks at lower temperatures. The same trend was observed for the addition of BC in hemp-PLA composites, but results were less marked since the addition of hemp fibers counterbalanced this effect by slightly increasing the T_cc_.

Regarding the melting phase, thermograms of PLA and PLA charged with 5% BC presented a single peak, whereas a double melting peak appeared when BC loading was beyond 5 wt% (Figure 4). Arrigo et al. [34] observed the double melting peak when BC exceeded 1 wt% loading in PLA. They proposed that the double melting peak could be explained by two different phenomena. The lamellar thickness model ([42,43]) suggests that two types of crystal lamellae with different thicknesses are present. Thus, the first endothermic peak corresponds to the melting of the thinner lamellae, while the thicker lamellae would melt at a higher temperature resulting in a second endothermic peak. The melting-recrystallization model ([44,45]) proposes that the melting of the existent lamellae is responsible for the appearance of the first peak, and these melted lamellae will then tend to recrystallize to form more organized lamellae which would need a higher temperature to melt and in turn generate a second peak. In our study, all the hemp-PLA composites also presented a double melting behavior. Similarly, Zhu et al. [46] observed one melting peak for PLA while PLA-sisal fibers composites presented two peaks. They explained that due to the complex crystallization process of polymers, the melting of different crystals type would happen differently during the heating and melting phases. Indeed, in the case of polymeric composites, transverse crystals may appear on the surface of the fillers when mixed with the semi-crystalline polymeric matrix and result in a second peak [47]. This explanation is applicable to justify our findings. Moreover, in our work, an additional separated peak appeared before the melting peak for PLA−20% BC and for the hemp-PLA composites (Figure 4). This peak may be attributed to poor blending of the fillers inside the PLA with the addition of fibers or a sufficient amount of BC. T_m_ values decreased when BC was added to pure PLA, which indicates that BC acted as a catalyst for the melting of the polymeric matrix to the detriment of the thermal stability of the samples. Regarding hemp-PLA composites with and without BC, slightly higher T_m_ was found as compared to neat PLA. These results are in accordance with those of TGA.

During the cooling cycle, the addition of BC to both PLA and hemp-PLA composites generated a slight increase in the T_c_ (Table 6), indicating that BC addition retarded the crystallization phase by limiting the connections between PLA chains. Furthermore, the crystallinity degree (X_c_) fluctuated differently from one sample to another. Overall, most of the samples had a smaller X_c_ compared to neat PLA except for the two hemp-PLA composites with 5 and 10 wt% BC that had higher X_c_. In general, molecular chains are rearranged during the crystallization process. PLA*’*s molecular chain integrity was disrupted after the incorporation of filler which influenced the mobility of PLA molecules and led to a change in the crystallinity degree [39,48,49]. In addition, the decrease in crystallinity might be related to the degradation and re-crystallization of polymer chains during the composites*’* preparation process (i.e., during extrusion and injection molding) [39]. For hybrid samples containing both BC and hemp fibers, the X_c_ was higher compared to neat PLA. The same phenomenon was observed previously in PLA-wood flour composites when glass fibers were added, as the results indicated a filler-matrix interaction on the interface of the crystal layer [39].

### 3.5. Hygroscopic Properties

#### 3.5.1. Water Contact Angle Measurements

The wetting behavior of the PLA-based composites was assessed by water contact angle (WCA) measurements which were recorded over a period of 30 s. Results are used to describe the water repellency ability of the studied samples. Good reproducibility of the WCA measurements was observed between the ten drops deposited separately with 10 mm spacing, relating a uniform surface of the composites. The WCA remained constant for all the samples from the deposition of the drop until the end of the test. For comparison, the WCA of PLA and hemp-PLA composites after 30 s were reported in Table 7.

For PLA-BC samples, the WCA decreased gradually by 3.3%, 4.3%, and 40% after incorporation of 5 wt%, 10 wt%, and 20 wt% of BC, respectively. Specifically, loading up to 20 wt% BC changed the composite nature from hydrophobic (WCA > 90°) to hydrophilic (WCA < 90°), according to Law*’*s definitions [50]. Generally, BC*’*s wettability is governed mainly by two key parameters: the origin feedstock and the carbonization temperature. Kinney et al. [51] investigated the changes in hydrophobicity of BC derived from three different biomasses (Magnolia leaf, corn stover, and applewood) prepared with temperatures ranging from 300 °C to 700 °C. They observed that BC*’*s hydrophobicity diminished proportionally as the pyrolysis temperature increased, with minimal hydrophobicity being correlated with BC prepared at temperatures between 400 °C and 600 °C. In our study, the BC was prepared at 550 °C, which could have resulted in a more hydrophilic BC. Therefore, when 20 wt% was added to PLA, the surface of the composite changed to be more hydrophilic.

The addition of hemp fibers in PLA lowered the WCA by 10.7% compared to neat PLA control. The hydrophilic character of natural fibers attributed to the presence of polar groups (namely hydroxyl groups) is responsible for the drop of WCA at the surface of the composite. Similar results were observed previously for Eucalyptus fibers reinforced-PLA [52]. Hemp fibers were characterized by high cellulose and hemicelluloses content [10], which give them a hydrophilic character. In general, polysaccharides are known to influence surface properties when used in polymeric composites as fillers; one of their main effects is to lessen the water repellency of the materials. Despite this decrease caused by hemp fibers, the value of WCA obtained in this study (up to 109°) is still higher compared to the 85° reported in prior research [41] for PLA reinforced by 30 wt% distiller*’*s grain (a by-product from ethanol fuel industries) fibers.

When 5 wt% BC was added to the hemp-PLA sample, the WCA increased slightly by 4.6%, which indicates that BC tended to stabilize the interaction between PLA and hemp fibers polymeric chains and helped to maintain a compact structure with tight connection by making the polar groups less available on the surface of the composites. BC might cancel the repulsion forces at the interface between hemp fibers and PLA, which led to a more compact and tight structure and enhanced the hydrophobicity. However, further increase in BC loading up to 10 wt% decreased the surface WCA.

E_wet_ corresponds to the energy required to wet the material. The wetting energy provides a quantitative measure of the intermolecular forces occurring at the surface of a material in the presence of a liquid. E_wet_ tends to be higher in the case of more wettable composites (i.e., hydrophilic composites). Based on our results (Table 7), we found that samples with lower WCA had higher E_wet,_ which agrees with the wetting law principles stating that when energy on the interface is low, the wettability (i.e., wetting) of a liquid on the surface is less favorable, which in turn will result in higher hydrophobicity [53].

#### 3.5.2. Water Absorption Measurements

The results collected from the water absorption immersion test are shown in Table 7. The reported values correspond to the averages from three repetitions per sample. Overall, all the specimens gradually absorbed water. The water uptake rate was faster during the first 24 h then was reduced as the composites retained more water.

Compared to the reference PLA, the WA after 24 h increased steadily with the increase of BC content by 51%, 110%, and 139% for 5, 10, and 20 wt% BC, respectively. Similarly, by including the hemp fibers, the water uptake dramatically increased. In fact, for the hemp-PLA specimens, the WA increased by 312%. Similar findings were also found by Silva et al. [52], who commented that the incorporation of eucalyptus fibers in PLA led to an increment in the overall permeability and diffusion velocity of water molecules in the studied composites. Chen et al. [41] explained the increase of the WA rate in the PLA distiller*’*s grain fibers composites with the occurrence of hydroxyl groups in natural polymers (i.e., cellulose, hemicellulose, and lignin). Finally, the WA of hemp-PLA continued to increase as the BC was added. These results can be assigned to the hydrophilic nature of the used BC that was discussed previously in the WCA section.

In the same context, Kumar et al. [23] investigated the effect of BC filler derived from bamboo and coconut shell biomasses on the properties of PP-bamboo fibers composites. They found that the increase in water uptake is mainly due to the ability of BC and plant fibers to absorb water. In the same study [23], the effect of pyrolysis temperature at which BC was prepared was evaluated, and the results indicated that for an equal loading of filler, BC carbonized at low temperature (300 °C) was correlated with lower WA percentage. BC prepared at higher temperatures often exhibits higher porosity which contributes to increasing the water uptake. The WA results found in our study are further confirmed by the information from physisorption analysis (Table 2) that informed about the developed porosity of our BC powder. Indeed, the presence of pores and cavities in the BC particles can facilitate water diffusion from the outside environment into the composites acting as transport channels. Another explanation was provided by Eselini et al. [54], who observed an increased WA for PLA reinforced with 30 wt% flax fiber. They reported that the lack of adhesion between the matrix and the filler contributed to the formation of micro-voids in the internal structure of the composite responsible for increasing the water uptake. In a similar experiment [41], the increase of WA rate in PLA-grain fibers composites was explained by the occurrence of hydroxyl groups in natural polymers (i.e., cellulose, hemicellulose, and lignin).

Overall, the results from the immersion test are in accordance with the results of WCA analysis. BC addition tended to reduce the water resistance of PLA and hemp-PLA composites. The use of BC produced at lower temperatures with higher hydrophobicity and lower porosity could be more suitable to improve the water resistance.

## 4. Conclusions

At present, research on polymeric bio-composites is experiencing rapid growth. In this present study, the color, mechanical, thermal, and hygroscopic features of biochar (BC) reinforced PLA and hemp-PLA composites were examined. The tensile test results showed that BC could improve the tensile modulus of elasticity and tensile strength of both PLA and hemp-PLA composites when added at 5 wt% loadings. Nonetheless, more than 5 wt% of BC resulted in lower mechanical strength compared to the reference samples due to the poor dispersion as BC particles usually tend to agglomerate. The uniform dispersion of the filler in the polymeric matrix is a key requirement to be fulfilled as it directly influences the strength of the materials. The TGA and DSC isotherms showed that BC slightly decreased the thermal stability by increasing the degradation of PLA polymers due to the BC’s catalytic effect. Moreover, the crystallization of PLA was hindered by the presence of BC. Lastly, BC lowered the water repellency of PLA and hemp-PLA composites, which was manifested by a decrease in hydrophobicity and an increase in the water absorbance. The decrease in water repellency was mainly attributed to the hydrophilic character and developed porosity of the used BC.

In view of limiting the price of PLA by introducing fillers while maintaining satisfying overall performance of PLA composites, biochar (BC) is an interesting candidate when introduced for up to 5 wt% of PLA or hemp/PLA in the case of bio-composites production. An optimization of BC properties to specifically target the composite applications, i.e., increasing hydrophobicity and decreasing porosity, could help improve the water repellency and potentially facilitate the dispersion of the fillers that was penalizing the mechanical performance of composites. Moreover, it might be of interest to consider using a compatibilizer when preparing the composites as it could promote better matrix-filler interaction.

## Figures and Tables

**Table 1:** Formulations of the prepared PLA-based composites.

Sample	PLA (wt%)	Hemp Fibers (wt%)	Biochar (wt%)
PLA	100	0	0
PLA-5% BC	95	0	5
PLA-10% BC	90	0	10
PLA-20% BC	80	0	20
PLA-30% HF	70	30	0
PLA-30% HF-5% BC	65	30	5
PLA-30% HF-10% BC	60	30	10

**Table 2:** Characteristics of beechwood biochar used for PLA reinforcement.

	Proximate Analysis				Particles Size Analysis		
Parameters	^1^ M, %	^2^ V, %	Ash, %	^3^ FC, %	D_10_, µm	D_50_, µm	D_90_, µm
Results	4.1 ± 0.1	16.3 ± 0.2	1.8 ± 0.1	77.8 ± 0.3	11 ± 1	60 ± 7	155 ± 5
Physisorption gas			^4^ SA, m^2^/g		^5^ TPV, cc/g	^6^ PW, nm	
Nitrogen			230.8		0.131	2.351	
CO_2_			480.0		0.133	0.349	

^1^ M: Moisture, ^2^ V: Volatiles, ^3^ FC: Fixed Carbon ^4^ SA: surface area, ^5^ TVP: total pores volume, ^6^ PW: average pores width.

**Table 3:** Surface color analysis results of PLA-based composites according to the CIELAB color system (CIE L*, CIE a* and CIE b* coordinates).

Sample	CIE L*	CIE a*	CIE b*
PLA	90.3 ± 0.4	−0.4 ± 0.0	4.8 ± 0.1
PLA-5% BC	27.8 ± 0.8	−0.5 ± 0.1	−6.7 ± 0.1
PLA-10% BC	24.2 ± 0.7	−0.4 ± 0.1	−4.6 ± 0.3
PLA-20% BC	13.8 ± 0.5	0.1 ± 0.0	−0.6 ± 0.3
PLA-30% HF	40.0 ± 2.5	2.9 ± 1.0	7.7 ± 2.7
PLA-30% HF-5% BC	21.2 ± 1.2	−0.1 ± 0.0	−1.5 ± 0.6
PLA-30% HF-10% BC	16.4 ± 0.3	−0.2 ± 0.0	−1.9 ± 0.1

**Table 4:** Tensile properties of the PLA-based composite specimens.

Sample	Tensile Modulus of Elasticity, MPa	Tensile Strength, MPa	Strain at Break, %
PLA	2418 ± 237	39 ± 5	6.8 ± 0.7
PLA-5% BC	3334 ± 256	37 ± 3	2.7 ± 0.3
PLA-10% BC	2649 ± 648	31 ± 6	1.8 ± 0.3
PLA-20% BC	2269 ± 115	16 ± 7	0.8 ± 0.3
PLA-30% HF	5158 ± 652	44 ± 1	2.0 ± 0.1
PLA-30% HF-5% BC	5841 ± 792	49 ± 5	2.1 ± 0.1
PLA-30% HF-10% BC	4992 ± 757	51 ± 0.4	2.0 ± 0.3

**Table 5:** TGA analysis results of PLA-based composites.

**Sample**	**^1^** **T_5%_, °C**	**^2^** **T_10%_, °C**	**^3^** **T_max_, °C**	**Residue_500 °C_, %**
PLA	301.36 ± 6.38	312.88 ± 5.58	335.93 ± 0.47	3.92 ± 0.20
PLA-5% BC	297.76 ± 2.26	308.34 ± 2.57	331.68 ± 0.86	8.35 ± 0.11
PLA-10% BC	297.08 ± 5.69	304.86 ± 6.00	329.24 ± 0.71	13.15 ± 0.12
PLA-20% BC	289.76 ± 1.54	302.42 ± 1.68	323.70 ± 0.94	22.09 ± 0.04
PLA-30% HF	314.58 ± 13.63	328.31 ± 8.92	352.55 ± 1.64	8.54 ± 0.02
PLA-30% HF-5% BC	318.99 ± 2.59	330.34 ± 1.36	351.08 ± 0.63	11.72 ± 0.48
PLA-30% HF-10% BC	311.71 ± 1.98	327.00 ± 0.75	349.66 ± 1.49	15.21 ± 0.04

^1^ T_5%_: Temperature at 5% weight loss, ^2^ T_10%_: Temperature at 10% weight loss, ^3^ T_max_: Temperature at maximum degradation rate.

**Table 6:** DSC analysis results of PLA-based composites.

Sample	T_g_, °C	T_cc_, °C	T_m_, °C	T_c_, °C	X_c_, %
PLA	58.8 ± 0.3	121.1 ± 0.8	149.6 ± 0.2	83.4 ± 0.1	5.7 ± 0.1
PLA-5% BC	59.3 ± 0.3	116.6 ± 0.4	149.1 ± 0.4	84.9 ± 0.2	2.5 ± 0.1
PLA-10% BC	57.7 ± 0.9	112.8 ± 0.9	147.1 ± 0.5	84.9 ± 0.1	4.1 ± 0.0
PLA-20% BC	52.9 ± 0.2	107.8 ± 0.3	146.5 ± 0.7	84.3 ± 0.1	4.5 ± 0.1
PLA-30% HF	58.6 ± 0.3	125.5 ± 0.4	150.3 ± 0.1	86.5 ± 0.1	4.1 ± 0.0
PLA-30% HF-5% BC	58.7 ± 0.1	121.5 ± 0.5	150.0 ± 0.2	87.6 ± 0.2	10.9 ± 0.2
PLA-30% HF-10% BC	58.6 ± 0.1	121.6 ± 1.0	150.0 ± 0.1	87.2 ± 0.1	6.7 ± 0.1

**Table 7:** Water contact angle, wetting energy, and water absorption results of PLA-based composites.

Sample	WCA, °	E_wet_, mJ/m^2^	Water Absorption, %	
	_	_	24 h	48 h
PLA	122 ± 4	5 ± 0	0.4 ± 0.0	0.6 ± 0.1
PLA-5% BC	118 ± 2	22 ± 3	0.6 ± 0.1	0.8 ± 0.0
PLA-10% BC	117 ± 2	29 ± 2	0.9 ± 0.3	1.1 ± 0.3
PLA-20% BC	73 ± 3	43 ± 2	1.4 ± 0.2	1.9 ± 0.1
PLA-30% HF	109 ± 2	6 ± 1	1.7 ± 0.0	2.3 ± 0.1
PLA-30% HF-5% BC	114 ± 3	3 ± 0	1.5 ± 0.0	2.1 ± 0.1
PLA-30% HF-10% BC	107 ± 3	10 ± 1	1.8 ± 0.0	3.5 ± 0.0

## Data Availability

Not applicable.
